# Determinants of successful mrsa decolonization among patients included in a clinical trial of polyhexanide

**DOI:** 10.1186/2047-2994-4-S1-P192

**Published:** 2015-06-16

**Authors:** E von Dach, C Landelle, T Haustein, A Agostinho, A Renzoni, P François, G Renzi, J Schrenzel, D Pittet, S Harbarth

**Affiliations:** 1 Infection Control Program, Univ. Hospital, Geneva, Switzerland; 2 Microbiology Laboratory, Univ. Hospital, Geneva, Switzerland

## Introduction

Between January 2011 and July 2014, we performed a Randomized Controlled Trial (RCT) in carriers of methicillin-resistant *Staphylococcus aureus* (MRSA) to test the efficacy of polyhexanide versus a placebo solution for MRSA decolonization.

## Objectives

We assessed determinants associated with successful MRSA decolonization among patients included in this RCT.

## Methods

We conducted a retrospective, unmatched case-control study among MRSA-positive patients treated by polyhexanide or placebo and a MRSA screening swab at day 28 after the end of the treatment. Cases were defined as patients with a negative MRSA screening at the end of the study. The control group consisted of patients still MRSA-positive at the end of the study. We tested potential promoting factors using univariate logistic regression analysis. Data were recorded prospectively during the initial RCT with the consent of the patients. A multivariate logistic regression model was then built. We kept as potential candidates for the multivariate analysis promoting factors with a p-value < 0.2. The resulting model was verified using the Hosmer-Lemeshow test.

## Results

A total of 135 patients were identified: 46 MRSA-negative cases (34.1%) and 89 MRSA-positive controls (65.9%) at end of follow-up. Cases were younger (Odds Ratio (OR) per 1-year increment; 0.98, P=0.079), lived without assistance (OR 2.97, P=0.005), had only the nose or groin colonized (OR 2.30, P=0.023), had no invasive devices (OR 2.53, P=0.065) and a length of hospital stay until first day of decolonization treatment of ≤ 3 weeks (OR 2.42, P=0.04), were more likely to be without malignancy (OR 2.66, P=0.073) or COPD (OR 2.23, P=0.119) compared to the control group. By multivariate analysis, two independent factors were associated with successful MRSA decolonization: independent status (OR 2.83, 95%CI [1.26-6.34], P=0.011) and only one colonized body site at baseline (OR 2.16, 95%CI [1.03-4.56], P=0.042; Hosmer-Lemeshow test, P=0.45).

## Conclusion

Independent patients with only 1 MRSA-positive body site at baseline (nose or groin) are more likely to be successfully decolonized.

This work was funded by B. Braun as an investigator-initiated research project.

## Disclosure of interest

E. von Dach: None declared, C. Landelle: None declared, T. Haustein: None declared, A. Agostinho: None declared, A. Renzoni: None declared, P. François: None declared, G. Renzi: None declared, J. Schrenzel Consultant for: bioMerieux, D. Pittet: None declared, S. Harbarth Grant/Research support from: a peer-reviewed research grant funded by Pfizer, Consultant for: the advisory boards of Destiny Pharma, bioMerieux, Novartis, and DaVolterra

